# Biomarkers in diagnosing and therapeutic monitoring of tuberculosis: a review

**DOI:** 10.1080/07853890.2024.2386030

**Published:** 2024-08-03

**Authors:** Sneha Leo, Meenakshi Narasimhan, Sridhar Rathinam, Antara Banerjee

**Affiliations:** a Department of Respiratory Medicine, Chettinad Hospital and Research Institute, Chettinad Academy of Research and Education, Kelambakkam, Tamil Nadu, India; b Faculty of Allied Health Sciences, Chettinad Hospital and Research Institute, Chettinad Academy of Research and Education, Kelambakkam, Tamil Nadu, India

**Keywords:** Biomarkers, tuberculosis, diagnosis, treatment monitoring

## Abstract

Tuberculosis (TB) continues to pose a significant health challenge worldwide, emphasizing the importance of prompt diagnosis and efficient monitoring of treatment outcomes for effective disease control. Biomarkers have become increasingly important in the realm of TB diagnoses and treatment. The objective of this comprehensive review is to examine the present state of biomarkers employed in the diagnosis of TB, monitoring the response to treatment, and predicting treatment outcomes. In this study, we undertake a comprehensive examination of the diverse biomarkers utilized in TB diagnoses, spanning molecular, immunological, and other novel methodologies. Furthermore, we examine the potential of biomarkers in the context of therapeutic monitoring, assessment of treatment effectiveness, and anticipation of drug resistance. Additionally, this paper presents future prospects regarding the utilization of biomarkers in the therapy of tuberculosis.

## Introduction

Tuberculosis (TB) is a significant contributor to global morbidity and mortality, resulting in approximately 10.6 million cases and 1.6 million fatalities in the year 2022^1^. Despite the presence of newer drugs, notable progress in TB diagnostics, and the implementation of National TB policies, the elimination of TB appears preposterous, especially in low and middle-income countries like India. Although TB is preventable and treatable, the COVID-19 outbreak has caused considerable setbacks. Interruptions in health services have resulted in projected increases in TB incidence of 5–15% over the next five years, as indicated by computational models [1]. A significant obstacle in the field is the absence of diagnostic tools possessing both high sensitivity and specificity, which can be effectively employed in Point-Of-Care (POC) facilities. Addressing this issue makes it possible to prevent patient attrition during the initial stage of the care cascade. It is widely acknowledged that early diagnosis of TB and prompt initiation of treatment are crucial elements in curbing the transmission of the disease within the community. These acts are integral to the overall strategy aimed at ending TB [2].

The sensitivity of standard TB diagnostic procedures, such as microscopy, is quite low, whereas the culture approaches are known to be time-consuming. The utilization of rapid molecular technologies, such as GeneXpert, is hindered by their high cost and limited accessibility in peripheral areas. In certain scenarios like smear negative Pulmonary Tuberculosis (PTB) and Extrapulmonary Tuberculosis (EPTB), the acquisition of suitable samples for molecular testing or culture can be challenging and necessitates invasive diagnostics techniques.

Biomarkers are characterized as objective and measurable attributes of biological processes [3]. A biosignature refers to a collection of biomarkers that serve as indicator for a distinct biological state or condition. The enhancement of support for biomarker research and development in the END-TB strategy has been advocated by the WHO [4]. An optimal TB diagnostic biomarker should possess the characteristics of practicality, requiring minimal instrumentation, and should be capable of utilizing readily accessible samples such as blood or urine. Extensive research has been conducted on both pathogen and host biomarkers, Table 1. Pathogen-derived biomarkers encompass the detection of both Mycobacterium tuberculosis (MTB) DNA and antigens. There is a diverse range of host-based biomarkers available, encompassing hematologic indicators, antibody response to antigens, cytokines and chemokines, RNA, other proteins, metabolites, and composite signatures incorporating numerous markers. The utilization of these biomarkers can be categorized based on their efficiency as diagnostic tests, and treatment response markers that have the potential to predict TB treatment outcomes. Therefore, the primary objective of this review is to provide a comprehensive description of the biomarkers utilized for the diagnosis of TB and for evaluating the effectiveness of therapeutic interventions in TB patients. The secondary aim of this study is to ascertain biosignatures that can be utilized for the development of POC diagnostic techniques for the detection of TB.

**Table 1.** Various platforms for detection of host biomarkers in TB.

	Sample type	Diagnosis	Platform	Specificity (%)	Sensitivity (%)	Reference
Host genes						
Neutrophil-driven type 1 interferon, interferon-γ, type 1 interferon-α or interferon-β gene signatures	Blood	Healthy controls, TB, LTBI	Illumina microarray, RT-qPCR validation	93.7, 96.6, 83	61.7, 94.1, 92	[5]
CD64 (FCGR1A), FCGR1B, GBP5, LTF, and GZMA	Blood	TB, LTBI	Agilent microarray	97	94	[6]
CD64 (FCGR1A), GBP5, and GZMA	Blood	TB, Non-tuberculosis pneumonia, Asthma	RT-qPCR	95	93	[7]
Gene signature	Blood	TB, LTBI, other diseases	Illumina microarray	96	100	[8]
Gene signature (51 genes)	Blood	TB, other lung diseases	Illumina microarray	83.6	82.9	[9]
CD64 (FCGR1A)		TB	MLPA technique	75	88	[10]
Gene signature (16 genes)	Blood	TB progression	Illumina RNA sequencing	82.8	53.7	[7]
Gene signature (144 genes)	Blood	Healthy control, TB, lung cancer, pneumonia	Illumina microarray	90	80	[11]
Metabolomic markers						
Ketone bodies, lactate, pyruvate metabolites	Plasma	Healthy controls, TB, pneumonia, diabetes	NMR spectroscopy	NR	NR	[12]
Ceramide, cholesterol sulfate	Plasma	TB, pneumonia	UHPLC–ESIQTOFMS	>80	>70	[13]
Host protein markers						
CRP		TB, HIV positive patients	POC florescent scanner	72	89	[14]
SYWC, gelsolin, kallistatin, CC9, testican-2, aldolase-C	Serum	TB	SOMAscan aptamer proteomics	80	90	[15]
CRP, IP10, transthyretin, interferon γ, SAA, CFH, ApoA1	Serum	TB, pulmonary diseases	Multiplex cytokine platform	73.3	93.8	[16]
Host microRNA						
hsa-miR-376c, hsa-miR-196b	Serum	Healthy control, TB, LTBI	Illumina sequencing	NR	NR	[17]
hsa-miR-150, hsa-miR-21, and hsa-miR-29c	Blood	Healthy control, TB, LTBI	microRNA microarray, RT-qPCR validation	88	91	[18]
miR-320a, miR-769-5p, and miR-22-3p	Plasma	TB, healthy controls	Illumina sequencing, RT-qPCR validation	NR	NR	[19]

### Target product profile criteria

WHO has established the performance and operational attributes of a test that is appropriate for primary care or at the POC settings through the utilization of its high-priority target product profiles (TPPs) criteria [20]. These criteria encompass the objective of achieving an optimal sensitivity of ≥ 98% in individuals with smear-positive, culture-positive pulmonary TB (PTB), a sensitivity level of at least 68% in smear-negative, culture-positive adults, and an overall pooled sensitivity of ≥ 80% in adults with HIV. It is advised to have a diagnostic sensitivity of at least 85% for lymph node-based extrapulmonary TB (EPTB) and at least 80% for cerebral spinal fluid in order to achieve good results in TPP testing. In the context of microbiologically confirmed TB, the objective is to achieve a TTP sensitivity of at least 66% specifically for intrathoracic TB cases occurring in infancy. A recommended objective for the sensitivity of the TTP in relation to these different phases of TB disease is to achieve a minimum value of 98%.

### Biomarkers for diagnosis currently in practice

#### Pathogen-based biomarkers

##### MTB-DNA

The GeneXpert MTB/RIF (2010) and GeneXpert MTB/RIF Ultra (2017) are nucleic acid amplification assays that utilize self-contained cartridges detect the presence of MTB DNA and assess resistance to the antibiotic Rifampicin by analysis of rpoB gene. These assays fall under the category of Catridge Based Nucleic Acid Amplification (CBNAAT) technology. The WHO has recently recommended the GeneXpert MTB/XDR (2021), a novel diagnostic test that enables the identification of resistance to Isoniazid and other second line anti-tubercular drugs, in addition to detecting MTB DNA. Although the sensitivity of TB detection is high (98%) in smear-positive samples, it is considerably lower in paucibacillary samples (68%). This is especially true for individuals living with HIV (80%), children (66%), and extrapulmonary specimens (79%) [7, 21,22]. The sensitivity and specificity profile for TB diagnosis is quite commendable. However, the GeneXpert machine and associated consumables are expensive and requires infrastructure with constant power supply. TrueNAT^TM^ is a POC diagnostic test that operates on battery power and using real time Polymerase Chain Reaction (PCR) technology. It has been endorsed by WHO as a reliable method for detecting MTB and Rifampicin resistance. The accuracy of the TrueNAT^TM^ is comparable to that of GeneXpert MTB/RIF [9]. It possesses the added benefit of being equipped with a battery-powered device, hence necessitating less infrastructure.

The utilization of MTB DNA as a means of diagnosis has been extensively employed for disease identification from a pathogen standpoint. The GeneXpert system has demonstrated the ability to detect MTB in several clinical samples, such as tissues, urine, and cerebrospinal fluid (CSF), with higher levels of sensitivity and specificity when compared to traditional MTB culture methods [13, 23–25].

##### Lipoarabinomannan

Lipoarabinomannan (LAM) is a prominent component of the cell wall/envelope of MTB, comprising approximately 15% of the bacterial weight. The architecture consists of a mannan "core" that is an expansion of the PIM structure, and this core is adorned with a solitary arabinan chain that exhibits branching. Furthermore, in the context of M. tuberculosis, a significant proportion of the final arabinose units in the arabinan chain are associated with small oligosaccharides comprising 2–4 mannose units, commonly referred to as "mannose capping motifs." This particular variant of LAM is often referred to as ManLAM.

LAM demonstrates significant and unique immunomodulatory characteristics. The factor in question is widely recognized as a virulence factor that is closely linked to the onset of MTB infection. In the context of mycobacterial infections, the presence of LAM in bodily fluids serves as a potential biomarker for the identification of individuals who are infected. Furthermore, it is worth noting that the immune reaction to LAM could potentially be utilized as a tool for diagnosis.

##### The identification of LAM in bodily fluids as a diagnostic approach for TB detection

The application of tests that identify MTB antigenic molecules in clinical specimens has the potential to provide numerous benefits in terms of a distinct biomarker for tuberculosis [26]. The tests conducted to detect urinary LAM have garnered significant interest and have been the subject of numerous academic publications. Nevertheless, numerous challenges have been encountered in the past years, leading to the prevailing agreement that the current diagnostic test(s) lack the necessary sensitivity to accurately detect latent or active TB in individuals who are not infected with HIV [27–30]. The presence of LAM has been demonstrated in the urine of people with active TB, as well as in rodents that were administered with sonicated MTB. In their study, Kamra et al. utilized a nano-based immuno-PCR (I-PCR) assay, specifically the magnetic bead-coupled gold nanoparticle-based I-PCR (MB-AuNP-I-PCR), to identify a combination of MTB LAM and MPT-64 markers within extracellular vesicles (EVs) found in the urine of patients with genitourinary TB (GUTB). The researchers then compared the outcomes of this assay with those of both I-PCR and Magneto-ELISA techniques [31].

In a randomized trial comprising 2600 hospitalized individuals diagnosed with HIV and TB, the incorporation of urine LAM-based evaluation through the utilization of the Alere TB-LAM test resulted in a noteworthy decrease in mortality among three specific segments of patients: those with diminished CD4+ T-cell numbers, profound anemia, or clinically presumed TB [32]. It was hypothesized that the levels of LAM in the urine of individuals with severe HIV are elevated and can be detected. This elevation is believed to be influenced by a significant burden of bacteria and the widespread dissemination of MTB throughout the body [33]. It appears plausible that individuals afflicted with a more localized form of TB would exhibit a reduced excretion of LAM.

WHO recommends the use of Lateral Flow LAM Assay (LF-LAM) for the diagnosis of active TB in people living with HIV in those with signs and symptoms of TB (or) with advanced HIV diseases (or) seriously ill (or) CD4 count < 200 cells/mm^3^ (IP settings) or < 100 cells/mm^3^ (OP settings) irrespective of signs and symptoms of TB [34].

##### Host immune reactions to LAM as potential biomarkers of TB

The selection of the source of LAM utilized for *in vitro* experiments holds significant value in accurately dissecting how the immune system reacts to LAM. The ManLAM molecule, obtained from both the laboratory MTB strain (H37Rv) as well as the clinical strains, has been observed to trigger proinflammatory cytokines in the macrophages and dendritic cells, among others [35–37]. In contrast, ManLAM obtained from the experimental strain MTB Erdman elicits either a lack of response or only a weak response, characterized by a combination of cytokines that are both pro- and anti-inflammatory. The presence of phosphatidylinositol mannoside during the biosynthesis of ManLAM could potentially explain the observed diminished proinflammatory reaction in certain ManLAM preparations [37].

The majority of research on the adaptive immune reaction to M. tuberculosis has primarily examined the reaction to antigenic proteins/peptides. However, there is still a limited investigation into mycobacterial antigenic substances with carbohydrate characteristics, despite the fact that lipoglycans/glycolipids are the predominant structural components of the mycobacterial cell wall and exert significant and unique influences on the immune response. The liposomal administration of LAM has been demonstrated to initiate the activation of T lymphocytes unique to MTB [38]. Recent findings indicate that mycobacterial glycolipids, particularly LAM, cause the production of poly cytotoxic T-cells in bronchiolar lavage, which has been related to defense from MTB infection [39].

##### Anti-LAM antibodies as TB biomarkers

The production of anti-LAM antibodies occurs in response to both TB infection and BCG vaccination [40–42]. The presence of these antibodies has been linked to the process of MTB opsonization and the limitation of intracellular proliferation [41,42]. Defense in mice was demonstrated through the indirect transfer of monoclonal antibodies against LAM [43]. Several commercial tests were developed from the data mentioned; however, none of them demonstrated sufficient specificity and sensitivity [44]. The limited number of investigations conducted utilizing the Mycodot test has demonstrated a notable specificity when compared to serological tests targeting different protein antigens. However, the sensitivity of the Mycodot test appears to be comparatively poor. While individuals infected with active TB were observed to generate antibodies that exhibit a diminished binding capacity to the outermost layer of live MTB and possess a low IgG/IgM ratio, there exists supporting data indicating that these specific antibodies may potentially impede the spread of MTB [45].

#### Host-based biomarker

##### C-reactive protein

C-Reactive Protein (CRP) serves as an indicator of overall inflammation and can be assessed through POC tests performed on capillary blood collected *via* finger prick. The WHO has recognized CRP as a screening marker for TB in PLHIV with a designated threshold of >5mg/L. CRP is shown to have sensitivity of 84% while the specificity is low. However, a positive CRP test should instigate a confirmatory test for diagnosis of TB. The potential advantages of including CRP into the process of ruling out TB infection prior to initiating TPT among people living with HIV are being taken into consideration [46]. The study conducted by Yoon et al. [14] examined the efficacy of a point-of-care C-reactive protein (CRP) finger-prick testing as a diagnostic method for TB among people who are HIV positive and have CD4 levels equal to or less than 340 cells/ml. The study was conducted in Uganda and focused on individuals who were commencing anti-retroviral therapy (ART). The diagnostic accuracy of this test for culture-confirmed TB was determined to have 89% sensitivity and 72% specificity [47].

### Potential biomarkers in pipeline

#### Host biomarkers

The quantification of the immune response of the host serves as a supplementary approach to the identification of intact MTB or its components. The identification of host biomarkers in easily obtainable specimens such as blood from the peripheral veins, saliva, or urine would offer significant benefits in the detection of paucibacillary, and extrapulmonary diseases, or situations where the collection of sputum specimens is challenging, such as in pediatric patients. One characteristic of adaptive immunity is its ability to recognize and respond to particular antigens. This specificity enables the immune system to mount faster and more robust responses upon past exposure to antigens, facilitating the identification of specific MTB antigens by the measurement of immune responses. The quantification of circulatory antibodies for pathogenic components in *ex vivo* blood samples can be achieved through the utilization of ELISA or comparable assays, which serve as indicators of humoral immune system activity. However, the effectiveness of immunological commercial tests has been inadequate, prompting the World Health Organisation to issue a cautionary recommendation against the utilization of the presently accessible tests [48].

#### Blood-based markers

The Complete Blood Count (CBC) from peripheral venous blood sample along with differential count can be performed in every resource limited setting and is widely available in almost all healthcare settings. The utility of Neutrophil-to-Lymphocyte Ratio (NLR) is being widely explored in diagnosis of tuberculosis. Miyahara et al. showed that a high NLR can be used to predict the risk of active TB in HIV infected individuals [49]. However, NLR is an inflammatory marker that may be associated with other infectious diseases and cannot be considered specific for TB. A meta-analysis to evaluate the role NLR in TB showed that patients with TB had low NLR compared to patients with bacterial pneumonia [50]. Thus the utility of NLR and its cut off is controversial. Mycobacterium tuberculosis (MTB) infects predominantly the myeloid lineages cells thus leading to increases in monocytes compared to lymphocytes. Thus Monocyte-to-Lyphocyte ratio (MLR) is more specific for MTB. High MLR has shown to predict TB disease in children with HIV, adults with and without HIV and has high sensitivity and specificity [51]. Hence MLR is a potential biomarker that should be further researched for validation compared with microbiological markers.

#### Host gene expression as biomarker of TB

Numerous investigations have investigated the efficacy of host transcriptomic biosignatures as potential diagnostic indicators and biomarkers for predicting the likelihood of potential tuberculosis onset. The most intriguing findings thus far have been the detection of genome-wide transcriptomic biosignatures in blood as a whole. Certain research groups have employed a combination of transcriptome and proteomic methodologies in order to investigate the progression toward active TB illness [52]. Berry et al. discovered the existence of an 86-gene complete blood transcriptomic signature in MTB infection, which is primarily driven by neutrophils and governed by type I interferon [53]. The gene *FCGRIB*, known as Fc gamma receptor 1B, exhibited significant upregulation in individuals diagnosed with TB. When combined with four additional genes (*LTF, guanylate binding protein 5, CD64, and Granzyme A*), FCGRIB demonstrated the ability to effectively differentiate between TB disease and latent tuberculosis infection (LTBI) in small case-control investigations. These studies reported a high level of sensitivity (94%) and specificity (97%) for this gene combination [54].

In their study, Kaforou et al. examined the transcriptional biosignatures in the blood of people with active TB, LTBI, and other infections. Through their investigation, they were able to identify a 44-transcript signature that effectively differentiated culture-confirmed TB cases from other diseases. This signature demonstrated high sensitivity and specificity (100 and 96% respectively) in a case-control study [55]. In a related study, Laux da Costa et al. expanded upon the previous research conducted by Maertzdorf et al. They specifically focused on comparing the expression profiles of three genes – *guanylate binding protein 5, granzyme A, and CD64* – in blood specimens obtained from patients diagnosed with TB disease, non-tuberculosis pneumonia, or Asthma [56].

Sutherland et al. expanded upon the research conducted by Maertzdorf et al. by assessing the biomarkers discovered by Maertzdorf et al. as well as additional mRNA transcription signatures. This evaluation was performed on a sample of 523 study subjects from four distinct African countries. The RT-MLPA technique was employed for this purpose [57]. The utility of CD64 as an indicator for TB was proven, regardless of the presence of HIV infection or the specific study site [58]. In a separate investigation, the utilization of CD64 in conjunction with three additional genes demonstrated a diagnostic accuracy for TB disease, having a sensitivity and specificity of 88 and 75% respectively [59].

In a small-scale investigation, Bloom et al. conducted a comparison of bloodstream genome-wide transcriptomic patterns between patients diagnosed with TB and individuals diagnosed with pneumonia, sarcoidosis, and lung cancer, as well as healthy controls. While there was a notable degree of similarity in the transcriptomic signatures observed in TB and sarcoidosis subjects when compared to other patient categories, a total of 144 transcripts were able to effectively differentiate TB from other diseases, exhibiting a sensitivity greater than 80% and a specificity over 90% [60]. The study conducted by Anderson et al. involved the assessment of mRNA transcript fingerprints in children exhibiting symptoms suggestive of TB in South Africa, Malawi, and Kenya. The researchers then compared these transcript profiles with those of children diagnosed with LTBI as well as other disorders [61].

#### Host protein markers

Interferon-gamma release assays (IGRAs) demonstrate limited utility in high-incidence settings due to the elevated prevalence of LTBI and the tests’ failure to effectively differentiate between LTBI and active TB disease. Investigations have been conducted on host markers, in addition to interferon-gamma, that are generated in response to novel or alternate antigens of MTB. Despite the identification of several antigens and host indicators with promising characteristics [62], the utilization of tests relying on stimulation assays, which sometimes need an extended period of time (often overnight), did not justify the increased delay in obtaining results. Consequently, these tests were deemed unsuitable for point-of-care quick diagnostic applications.

The utilization of the SOMA scan technique has led to the identification of a six-protein biosignature. This biosignature has been found to be effective in detecting TB, exhibiting a sensitivity of 90% and a specificity of 80% across a sample size of over 700 individuals from diverse TB-endemic regions [63].

#### Metabolomic markers

Several analytical techniques, such as NMR spectroscopy, GC-TOFMS, and LC-MS, have been utilized for the identification of small metabolites that exhibit discriminatory capabilities between TB and other diseases. Despite the emergence of potential metabolites and pathways, the current body of research primarily consists of small-scale case-control studies. Consequently, the diagnostic efficacy of these proposed metabolites remains to be substantiated by comprehensive investigations including larger study cohorts. Zhou et al. employed NMR spectroscopy to examine variations in the blood metabolic identities of individuals diagnosed with TB disease and a control group of healthy individuals [57]. This study builds upon the previous research conducted by Weiner et al. who identified a number of distinct metabolites that differentiate TB cases, latent LTBI cases, and normal controls [57].

A total of 30 metabolites were discovered, with 17 of them exhibiting considerably increased expression levels in serum samples obtained from TB patients as compared to the control group [5]. In a subsequent investigation, researchers were able to discern that ketone bodies, lactate, and pyruvate are the metabolites that possess the greatest potential for distinguishing TB illness from other disorders such as community-acquired pneumonia, diabetes, and patients with different cancers [64]. Du Preez et al. conducted a proof-of-concept study wherein they utilized an undefined metabolomics technique to examine host molecules in sputum specimens. This investigation involved the use of two-dimensional GC-TOFMS.

In their study, Cho et al. employed a focused methodology that prioritized non-lipid metabolites. This technique enabled the identification of specific ratios between amino acids that exhibited discriminatory capabilities in distinguishing ATB, TB infection, and individuals without TB [8]. Conde et al. conducted an evaluation of amino-acid signatures and successfully identified a signature that aligns with the World Health Organization’s TPP guideline for a triage test aimed at ruling out active tuberculosis [6].

Despite the considerable variation observed in the metabolites detected across samples from various individuals, certain metabolites such as fatty acids, carbohydrates, and mycolic acids exhibited promise as prospective biomarkers [65]. Plasma samples are consistently revealing additional metabolic indicators, such as molecules implicated in glucose, lipid, and amino acid breakdown processes [10]. The investigations conducted thus far have yielded valuable insights into the metabolic reaction to MTB infection, thereby contributing to our comprehension of the intracellular survival mechanisms of MTB. However, further investigation is required to ascertain the diagnostic prospective of these methods for TB [11].

#### Proteomics

In the field of TB examinations, transcriptomic investigations that have successfully identified blood gene expression profiles with diagnostic usefulness are considered to be at the forefront of advancements. According to a previous systematic review and meta-analysis, it was discovered that 17 transcriptome signatures fulfilled a minimum of one performance criterion for TB diagnostics as outlined by the TPP. Furthermore, three of these signatures were verified in cohorts that were clinically relevant [66]. The transcriptome signature known as RISK11 demonstrated favorable functionality as a screening tool for symptomatic ATB [16, 67]. However, its efficacy in detecting asymptomatic (subclinical) tuberculosis was notably diminished [14].

A further investigation, which examined eight concise indicators, yielded comparable results, indicating that the ability to accurately diagnose symptomatic TB infection was generally excellent. However, the performance in identifying sub-clinical disease was notably inferior [68]. Given the significant occurrence of subclinical disease, it is imperative to enhance the diagnostic accuracy of subclinical TB in order to enhance the effectiveness of case identification efforts [15].

#### MicroRNAs

Numerous researchers have conducted assessments on the potential diagnostic use of miRNAs as biomarkers for TB [69]. The study conducted by Zhang et al. aimed to assess the serum miRNA signatures that can distinguish between active TB, LTBI, and healthy controls. The sample size consisted of 15 TB subjects and 82 controls. The researchers identified several miRNAs that exhibited either up-regulation or down-regulation in TB patients, specifically 24 and 6 miRNAs, respectively. Among these miRNAs, has-miR-196b and has-miR-376c demonstrated promise as potential diagnostic markers for TB following validation through RT-PCR. However, the study’s findings are constrained in their global practicality due to the restricted sample size and absence of a validation cohort [12].

Latorre et al. performed a study examining blood miRNA fingerprints in individuals with TB disease, LTBI, and healthy controls. The study utilized micro-arrays and confirmed the findings through RT-PCR. The results revealed a three-marker miRNA signature that effectively identified active TB disease, distinguishing it from LTBI controls. The diagnostic sensitivity of this signature was determined to be 91%, while the specificity reached 88% [70].

In a separate investigation, a total of 29 microRNAs (miRNAs) were identified as exhibiting differential expression patterns between individuals diagnosed with TB and control subjects. Notably, three of these miRNAs showed the ability to effectively distinguish between TB disease and the absence of the condition in healthy control patients, as confirmed through validation using RT-PCR. The area under the curve (AUC) values for these discriminating miRNAs ranged from 0.69 to 0.97. Validation of these small-scale case-control studies is necessary through larger cohort studies [71].

### Biosignatures

#### Host antibodies

Infection and immunity are two interrelated aspects of the same phenomenon. Upon infection with MTB, the microbe will unavoidably trigger the immunological function of the host, resulting in alterations in host biomarkers. Pathogen-specific antibodies serve as widely utilized host biomarkers in disease diagnostics due to their simplicity, cost-effectiveness, and suitability for point-of-care applications. The most researched subset of biomarkers is MTB antigen-specific antibodies, which can be used to diagnose extrapulmonary or pulmonary TB. Nevertheless, these tests have not demonstrated utility in distinguishing between asymptomatic MTB infection and active tuberculosis (ATB). Additionally, their diagnostic accuracy is compromised by their limited affinity for the surface antigen [72], leading to inconsistent sensitivity and specificity across multiple trials [73,74]. Several antibodies specific to MTB antigens, including PPD, CFP-10, ESAT-6, antigen 60, lipid-derived antigens, and heat shock protein(s), have been intensively investigated [75]. It is worth mentioning that certain antigens of MTB, such as ESAT-6 and CFP-10, are absent in the genetic makeup of the BCG strain. Consequently, the identification of an immune reaction targeting these antigens can serve as a means to differentiate between a MTB infection and a response to vaccination. Regrettably, the assays conducted thus far have exhibited suboptimal sensitivity, ranging from 14% to 85%, as well as specificity ranging from 53% to 98.7% [76].

According to the findings of Lopez-Ramos et al. the utilization of antibodies targeting the MTB antigen P12037, in conjunction with sCD14, demonstrated a sensitivity of 92% and a specificity of 91% in the accurate diagnosis of active TB [77]. Previous studies conducted by researchers have demonstrated that the presence of antibodies against specific antigens of MTB, such as proline-proline-glutamic acid protein 17 (PPE17) [78] and mycobacterial DNA binding protein (MDP-1) [79], might serve as a distinguishing factor between individuals with LTBI and those with active TB.

In their study, Maekura et al. demonstrated that MDBP-1 has potential as a screening tool. They found that consistently high levels of IgG antibodies against MDBP-1 following anti-TB medication could be indicative of a relapse occurring after the end of treatment [79]. However, it has been observed in several investigations that children generally exhibit low levels of antibody reactions against MTB antigens. Consequently, serological testing for the identification, prognosis, and therapeutic monitoring of TB can only be reliably employed among adult patients [80]. A notable investigation conducted by Lu et al. examined a group of individuals referred to as "resisters" who exhibited significant exposure to MTB, yet tested negative for both tuberculin skin test (TST) and T-cell-based IGRA and did not acquire LTBI [81]. This work has provided valuable insights into the pathophysiology of TB. The researchers discovered that the individuals classified as "resisters" had class-switched IgG and MTB-specific IgM. However, these individuals demonstrated diminished CD4-mediated IFN-γ responses when exposed to CFP-10, ESAT-6, Ag85A, and Ag85B [81].

#### TB-specific antibodies in saliva

In 1973, research carried out in the United States aimed to assess the concentrations of hemagglutinating IgA specific to tuberculosis polysaccharide and protein in saliva. Salivary antibodies were observed in a mere 5% of TB cases and chronic obstructive pulmonary disease (COPD) patients, based on a sample size of 20 individuals for each group. This finding suggests a notably low level of both specificity and sensitivity. Subsequent investigations yielded more encouraging findings. In their study, Araujo et al. utilized the ELISA technique to assess secretory IgA levels in Venezuelan children’s saliva samples. The specific target of interest was the 38 kDa antigen of MTB [17]. Raras et al. assessed secretory IgA levels by measuring antibodies against an engineered semi-purified antigen of 38 kDa in saliva samples obtained from 30 adult TB cases and 30 healthy controls in Indonesia. The researchers employed the dot blot technique for their analysis [18].

The sensitivity of anti-38 kDa antibodies in the bloodstream is generally high, however, their restricted sensitivity has hindered their prospective as serological examinations for diagnosing TB [19]. The effectiveness of saliva as an assessment tool does not exhibit superior performance. The screening efficiency of the anti-38 kDa antibodies in the saliva was found to be significantly below the criteria set by TPP in both studies.

#### Host: cytokines and chemokines

In contrast to antibody reactions, cellular defenses targeting MTB-specific antigens have demonstrated enhanced consistency. Historically, TST has been extensively employed for the purpose of diagnosing both active TB and LTBI. Nevertheless, the test’s inability to distinguish MTB and various other non-tuberculosis mycobacterial illnesses, along with the BCG vaccination, is attributed to cross-reactivity. In addition, it should be noted that the TST has limited sensitivity when administered to individuals with impaired immune systems. In recent years, the T-cell-based IGRA has gained significant prominence as a widely utilized method in TB diagnoses. The IGRA assay is employed to quantify the generation of interferon-gamma (IFN-γ) following the *ex vivo* stimulus of the whole blood with MTB-specific antigens, including CFP-10, and ESAT-6 [82,83]. In general, the IGRA has higher sensitivity and specificity compared to the TST [84].

While the IGRA test is less influenced by the HIV status in comparison to the TST [85], it demonstrates suboptimal performance in kids with progressed HIV infection [86]. However, when the IGRA test is conducted utilizing peripheral blood mononuclear cells (PBMC) obtained from specific sites of TB infection, including BAL, pleural fluid, and cerebrospinal fluid (CSF), it has been observed to exhibit high levels of sensitivity and specificity [87,88].

Several studies have indicated that the IGRA elicits a more robust immune response in individuals with active TB compared to those with LTBI. Consequently, this assay can be utilized to distinguish between the two kinds of TB disease [89]. Nevertheless, other research has indicated that the IGRA may not be appropriate for accurately diagnosing active TB and LTBI in countries with a high prevalence of TB [90]. Arroyo et al. conducted a study that demonstrated the potential for improvement in the utilization of latency-related antigens, specifically the resuscitation-promoting factor (Rpf) and dormancy survival regulon (DosR) in the context of IGRA. The peptides RV2029c (referred to as DosR peptide) and RV2389c (referred to as Rpf peptide) have demonstrated the ability to distinguish between LTBI and active pulmonary tuberculosis (PTB) having a sensitivity of 90% and 85% respectively [91].

Numerous investigations have been undertaken to employ multiplex cytokine bead arrays on plasma samples, both with and without *ex vivo* stimuli, in order to distinguish between active TB and LTBI. The studies utilize a collection of 5–15 biomarkers in their analyses, with sensitivities ranging from 82.3% to 96.7%.

### Drug resistance prediction

The prevalence of multidrug-resistant tuberculosis (MDR-TB) and extensively drug-resistant tuberculosis (XDR-TB) is escalating on a global scale. Ensuring timely access to efficacious treatment is crucial in order to mitigate the spread of infection and impede the development of drug resistance resulting from suboptimal therapy. Phenotypic culture-based approaches continue to serve as the primary approach for drug sensitivity testing in reference labs. However, these procedures are characterized by their time-consuming nature, often requiring several weeks to yield results and necessitating the implementation of rigorous safety measures in microbiology. The approaches employed in different laboratories exhibit a lack of consistency, resulting in variations. Furthermore, the relationship between microbial breakpoints and clinical efficacy for specific medications remains questionable [92].

Pharmacological resistance in MTB arises as a result of genetic alterations within the bacterial genome, which subsequently impact the efficacy of pharmacological targets or the functionality of supporting enzymes. Single nucleotide polymorphisms (SNPs) represent the most commonly detected kind of genetic variation. The detection of these little alterations in the sequence of DNA can be readily achieved by the process of amplification, offering a prompt and precise method for evaluating resistance to rifampicin. Line Probe Assays (LPA) are commercially accessible from multiple vendors. These assays involve the amplification of target areas using PCR, followed by the interrogation of the resulting amplicons using membrane-bound probes. LPAs can be utilized to determine the susceptibility of rifampicin and some other primary and second-line medicines.

Three technologies, namely Genedrive MTB/RIF, Cepheid Xpert^®^ Ultra, and Truenat MTB, were specifically developed to be utilized inside clinical settings equipped with microscopy facilities. The remaining technologies are anticipated to be employed in reference laboratories. The utilization of gene sequencing has witnessed a growing trend in the detection of treatment resistance in MTB due to its enhanced precision [92]. In an earlier comprehensive genome-wide association study (GWAS) focusing on MDR and XDR TB, it was observed that the ability to identify drug resistance to capreomycin, ethionamide, cycloserine, pyrazinamide, and para-aminosalicylic acid was significantly improved by incorporating deletions and insertions into the analysis [93]. This implies that the current method of SNP identification may not be sufficient for these medications, and it may be necessary to employ more advanced molecular devices.

The utilization of next-generation sequencing (NGS) and comprehensive genome analysis holds the potential to establish itself as the prevailing benchmark for the discovery of resistance to drugs in the future. The accessibility of NGS has significantly improved due to cost reductions, the creation of high throughput sequencing centers, and the availability of user-friendly analytical tools. As a result, NGS is now being widely adopted as a standard service in many nations. Nevertheless, in the majority of nations with a high disease burden, it continues to serve as a research instrument, with the analysis being conducted outside of the country. The feasibility of NGS straight from phlegm has been established as a proof of concept. However, the full evaluation of sputum samples requires the prior isolation and cultivation of the microorganisms in order to collect an adequate amount of bacterial DNA. This requirement poses a limitation to the practical use of NGS in patient care [94,95].

### Biomarkers in evaluating effectiveness of therapeutic interventions

The Sputum smear staining and assessment of sputum culture conversion, including both solid and liquid media, are traditionally employed to evaluate the effectiveness of TB treatment. However, it is worth noting that these approaches have limited sensitivity and typically requires a considerable amount of time, often spanning several weeks. The GeneXpert MTB/RIF assay provides quick detection in this context and has demonstrated favorable sensitivity (97%) and a correlation with culture positivity in terms of time. However, it is associated with suboptimal specificity, ranging from 49% to 72% [96,97].This is partially attributed to the utilization of GeneXpert, a PCR-based method that is capable of detecting the entirety of DNA found in the sample, regardless of whether the bacteria present are alive or deceased in clinical samples. Hence there is need for identifying biomarkers which can be utilised for rapid as well as reliable monitoring in TB treatment response.

## MTB RNA

The mycobacterial mRNA has a shorter life-span compared to DNA. The decline in mRNA concentration reflects decrease in MTB bacilli with the initiation of treatment. Thus mRNA based sputum assays are capable of differentiating viable from non-viable organisms. In a study conducted by Mdivani et al. continued presence of antigen 85B mRNA by PCR method correlated with failed response to treatment due to drug resistance [98]. Molecular Bacterial Load Assay (MBLA) by PCR method in sputum, to detect 16S RNA of MTB was shown to quantify viable bacterial load in sputum precisely. This method was comparable to MTB culture and accurate than DNA PCR and sputum microscopy in assessing the response to treatment [99].

### MTB antigens

The concentrations of MTB antigens like Ag85 and MPT64 in sputum, ESAT-6 and CFP-10 in serum were utilised to monitor therapeutic response in TB [100–102]. The reduced concentration or undetectable concentration reflected the efficacy of TB treatment. However extensive studies are needed to establish sensitivity and specificity of these methods comparable to standard methods.

### Host biomarkers

Cytokines are the most widely studied biomarkers for monitoring treatment response while chemokines are the least studied. Jacobs et al. observed significant decline in concentration of host markers like CRP, SAP, ferritin, IFN-γ, VEGF, IP-10, CC3, CFH and α-1-antitrypsin from baseline before initiation of therapy and significant rise in concentrations of transthyretin and MMP-2 [103]. In another study, the levels of TNF-α, IL6 and IL10 were significantly lower compared to baseline and IFN-γ and IL-4 were significantly higher after completing 2 months of ATT [104]. The levels of TNF-α and IFN-γ are inconsistent in different studies and needs further research.

### Blood-based markers

Choudhary et el observed that the MLR decline from baseline with initiation of treatment over a period of 6 months in children with HIV having Pulmonary Tuberculosis [105]. The same was observed in a cohort of adults with active tuberculosis in China. However, the utility of MLR in therapeutic response monitoring is yet to be validated.

### Limitations with the routine utility of biomarkers as diagnostic tool

TB manifests with a diverse range of clinical presentations and infectious features across various subpopulations. While there are numerous studies that have undergone internal validation, the external validity of biomarkers in relation to a diverse population remains uncertain.

- Though numerous studies are available on biomarkers, there nearly no biomarker that has equal or superior sensitivity and specificity to replace standard traditional diagnostic methods like microscopy, culture and histologic examination of specimens.
- The epidemiology of TB reveals that EPTB constitutes around 20% of the total number of active TB cases on a global scale. However, the majority of the investigations have focused on individuals with PTB, resulting in limited available information regarding EPTB.
- Pregnancy is characterized by a modified immunological response that includes immune suppression, leading to notable effects on the clinical manifestation of active tuberculosis. Insufficient research has been conducted within this particular section of the population.
- Pediatric TB poses a diagnostic challenge due to its often paucibacillary nature and inability to expectorate adequate sputum sample for routine testing. Research on biomarkers for TB in children has some limitations, such as the absence of validation in diverse populations and often small sample sizes. Additionally, the existing research often lack cases that have been verified through culture.
- TB-HIV coinfection is a challenging condition to detect due to its unusual presentation. The majority of TB biomarker studies are conducted on individuals who do not have HIV in order to assess their accuracy. However, it is important to note that the effectiveness of these biomarkers may differ in individuals with HIV depending on their immunological status. Therefore, it is imperative to conduct studies with a substantial sample size that encompasses many subgroups of individuals in order to authenticate the existing data.

Enhancing the efficacy of TB case identification and the identification of treatment resistance are key focal points for the WHO and the STOP-TB Alliance. Significant advancements have been achieved; yet, there are still unresolved study inquiries pertaining to the advancement of crucial diagnostic tests. The process of validating and evaluating novel technologies is currently underway, with additional testing planned for the future. The optimal test should possess a combination of elevated sensitivity and specificity; nevertheless, achieving this perfect balance may not always be feasible, necessitating the need for compromises.

In order for a new test to be considered satisfactory, it is imperative that its accuracy is at least equivalent to the existing tests. However, it is worth noting that lower sensitivity may be deemed acceptable for technologies that enhance the identification of cases, particularly in places where conventional clinic or laboratory-based medical services are not easily accessible. When considering the use of a test for initiating anti-tuberculosis therapy, it is crucial to prioritize high specificity. However, in situations where the device is intended for triaging patients or community-based screening, a lesser level of specificity may be deemed acceptable.

The WHO has released targeted product profiles that suggest a diagnostic test should ideally have a specificity of 98%. However, a minimum specificity of 70% is recommended if a follow-up confirmation test is planned, a minimum specificity of 70% is recommended. When creating novel biomarker-based tests, test developers encounter two significant hurdles. One potential limitation is that business pressures could potentially hinder the selection of the most optimal array of biomarkers. The act of obtaining patents for biomarkers is a widely adopted approach, and the desire to be the earliest entity to introduce a type of biomarker to the marketplace is a strong deterrent to collaborative efforts. One further obstacle is quantifying numerous indicators within a wide spectrum of concentrations, including some that are exceedingly low. The existing detection technologies are prohibitively costly and ill-suited for implementation at the point-of-care in regions with a significant tuberculosis prevalence.

In addition to the inherent biological and technical complexities associated with the development of innovative diagnostic instruments for TB, there exist significant operational, economic, and organizational obstacles that must be effectively addressed. The expenditure associated with the development of the devices may be surpassed by the financial outlay required for the preparation of a new market entrance test. In order to obtain regulatory authorization and endorsement from the WHO, it is imperative to conduct studies on test performance within groups that are representative of the intended users.

### Strengths of utilising biomarkers as diagnostic tool

Though the diagnostic methods using biomarkers are not yet validated and our knowledge gap in this field is still gigantic, in near future, biomarkers are expected to change our dimensions of approach towards TB diagnosis and TB control programmes. Advantages of using biomarkers are:

- Biomarkers will be able to identify TB disease at an earlier stage, even before development of symptoms, thus forming an integral part of TB screening programmes and aid in curtailing the enormous community spread.
- Biomarker tools will phase out the bio-hazardous risk associated with sample collection and processing of infectious samples like sputum.
- Diagnosis will be established from readily available sample like urine and blood, thus eliminating the need for obtaining invasive samples especially in smear negative PTB, EPTB and immuno-compromised population with paucibacillary TB.
- Methods will be established to devise biomarker kits that can be procured at a low cost and operated in resource limited settings. Thus these tools can be utilised in point-of-care platforms worldwide.
- Same biomarkers can be utilised for diagnosis of disease as well as monitoring therapeutic effectiveness at regular intervals.

## Conclusion

Despite significant progress made in the diagnostic tools and treatment methods, TB is still a serious global public health problem. Delayed diagnosis, poor treatment adherence and inadequate treatment have all kept the TB epidemic alive. The current strategy of relying solely on passive case finding for TB has been ineffective in containing the epidemic. It is now widely acknowledged that simply enhancing diagnostic technology will not be sufficient in curbing transmission, unless additional measures are put in place to facilitate early diagnosis and treatment monitoring. There is an urgent need for developing diagnostic methods that will facilitate rapid diagnosis of TB at an earlier stage, with high sensitivity and specificity, can be utilized to treatment monitoring and affordable to resource limited settings where the TB burden is more. The researches on biomarkers and bio-signatures are an assuring in meeting the above said needs and overcoming the shortfalls of conventional tools. However, the studies conducted are heterogenic and results are inconsistent. The practicality of these tools in diagnosing TB especially in EPTB, smear negative PTB and TB in high risk population are yet to be explored. Moving forward, large studies with large sample size, international cohorts, standardised research protocol and methodology, systematised laboratories and head on comparison with standard methods are needed to unveil the applicability of biomarkers in routine practice.

## Data Availability

Data sharing is not applicable to this article as no new data were created or analyzed in this study.
